# Awareness, attitudes and cost among patients for dental implants in teeth replacement

**DOI:** 10.6026/9732063002001358

**Published:** 2024-10-31

**Authors:** Maninderjit Kaur, Kirithana Chamakuri, Pranav Parashar, Charmee Hiten Shah, Pargat Singh, Layal Touchan

**Affiliations:** 1 Adesh Institute of Dental Sciences and Research Centre, General Dentist, Hoshiarpur, Punjab, India; 2 Government Dental College, General Dentist, Vijayawada, Andhra Pradesh, India; 3 Department of Dentistry, N.S.C.B Medical College and Hospital, Jabalpur, Madhya Pradesh, India; 4 AMC Dental College and Hospital, Ahmedabad, Gujarat India; 5 JCD dental college, General Dentist, Malikpur, Safidon, Jind, Haryana, India; 6 Aleppo University, Aleppo, Syria

**Keywords:** Dental implants, patient awareness, cost perception, attitudes, tooth replacement, implant education, affordability, treatment modality

## Abstract

Dental implants are increasingly viewed as a preferred treatment option for tooth replacement, yet gaps remain in patient knowledge, attitudes, and cost perceptions. This cross-sectional, *in vitro* study surveyed 150 adults aged 20-60 to assess their awareness, attitudes, and perceived costs of dental implants. Results show that while 70% of patients are aware of implants, only 30% understand the procedure. Though 60% expressed interest in implants, 45% perceived them as too expensive. Positive attitudes correlated with greater awareness, highlighting the need for enhanced education on the benefits and financial options associated with implants.

## Background:

Dental implants are increasingly becoming the standard for teeth replacement due to their long-term benefits, durability, and resemblance to natural teeth [1]. Unlike traditional options such as dentures or bridges, dental implants provide superior functionality, improved oral health and enhanced aesthetic outcomes [2, 3]. As a result, more patients and dental professionals are considering implants as the preferred treatment modality [4-6]. However, patient knowledge, attitudes and perceptions about dental implants still vary [7 -8]. Many patients lack awareness of the advantages of implants, often perceiving them as complex or unaffordable. Misconceptions regarding cost also play a significant role, as many individuals assume that implants are prohibitively expensive compared to traditional options without fully understanding the long-term value they provide in terms of reduced maintenance and better oral health outcomes [9]. Therefore, it is of interest to evaluate patients' awareness, attitudes and perceived costs of dental implants in comparison to other teeth replacement methods. It seeks to assess the extent of knowledge patients have about implants, their attitudes toward opting for them over other treatments, and how they perceive the financial aspect of this treatment. Understanding these factors is crucial for healthcare providers to tailor education and address concerns, ultimately enhancing the adoption of implants as a standard option for tooth replacement.

## Methodology:

This cross-sectional, *in vitro* study was conducted using a structured questionnaire with a sample size of 150 participants. The study focused on adults aged 20-60 who either required or had previously received teeth replacements. Participants with underlying health conditions that could prevent them from undergoing dental implant treatment were excluded. Data collection was carried out in dental clinics, with surveys designed to assess three critical areas: awareness, attitude and cost perception. The awareness section evaluated participants' knowledge of dental implants as a viable option for tooth replacement. The attitude section explored their willingness and openness to undergo the implant procedure, reflecting how comfortable and confident they felt about choosing implants over other treatments. Lastly, the cost perception aspect examined patients' views on the affordability and long-term value of dental implants, comparing their understanding of the financial investment against alternatives such as bridges or dentures. This structured approach provided a comprehensive understanding of how patient knowledge, attitudes and perceived costs influence the decision-making process regarding dental implants (Annexure 1 Survey Questionnaires).

## Results:

The study's results offer valuable insights into patient demographics, awareness, attitudes, and cost perceptions regarding dental implants. Among the 150 participants, 25% were aged 20-30, 35% were 31-40, 30% were 41-50, and 10% were 51-60. Males made up 60% of the sample, while females accounted for 40%. In terms of education, 40% had a high school education or lower, 45% were college-educated, and 15% held post-graduate degrees. In terms of awareness, 70% of the participants were aware of dental implants as a treatment option. However, only 40% believed that implants provided a permanent solution for tooth loss, and a mere 30% demonstrated a clear understanding of the implant process and necessary aftercare. Despite this limited understanding, interest in implants remained strong, with 60% of patients expressing interest in opting for the procedure. Notably, 50% preferred implants over alternatives such as bridges or dentures due to their longevity, though 35% expressed concerns about the surgical procedure and recovery process. Perceived cost was a significant factor for many participants. Nearly 45% of patients perceived dental implants as too expensive, while 30% were unaware of potential insurance coverage or instalment payment options that could make the procedure more accessible. Nonetheless, 25% of participants were willing to invest in implants, recognizing the long-term benefits and value compared to other tooth replacement modalities. These findings highlight the importance of improving patient education and addressing financial concerns to encourage greater adoption of dental implants (Figure 1 - see PDF).

## Explanation:

Here is the heatmap sketch for your survey results. The intensity of the colours reflects how participants responded to each category (Awareness, Attitude, and Cost Perception) based on different levels of response (from Highly Positive to Highly Negative). The darker green areas represent higher positive responses, while red indicates negative responses.

## How to read the heat map:

Rows represent survey categories (Awareness, attitude and cost perception).

Columns represent the intensity of responses (from highly positive to highly negative).

Colours (for the heatmap) should range from light (lower values) to dark (higher values)

Highly positive: Dark green (high percentage of positive responses).

Moderately positive: Light green.

Neutral: Yellow.

Negative: Light red.

Highly negative: Dark red (high percentage of negative responses).

This heat map gives a visual representation of how participants responded to the survey across various factors, making it easier to spot trends and areas needing focus.

## Discussion:

The study reveals key insights into patients' awareness, attitudes and perceptions regarding dental implants as a treatment modality for tooth replacement. While 70% of participants were aware of dental implants, significant gaps in understanding the procedure and post-treatment care were evident, with only 30% of participants demonstrating adequate knowledge. This aligns with previous studies, which have also found that while awareness of dental implants is generally high, detailed understanding of the procedure and aftercare remains limited among patients [10, 11 & 12]. A striking finding from this study is that 60% of patients showed interest in opting for implants, underscoring the growing recognition of implants as a superior option compared to bridges or dentures. However, the perceived high cost of implants emerged as a significant barrier, with 45% of participants considering implants too expensive. This reflects trends observed in the literature, where cost has consistently been identified as a major obstacle to the wider adoption of dental implants [13, 14]. Furthermore, 30% of the participants were unaware of insurance coverage or payment plans, indicating that educational efforts about financial assistance options could significantly impact patient decisions. Attitudinal shifts toward implants were also influenced by the level of awareness. Patients with better understanding of the benefits and longevity of implants demonstrated more positive attitudes, with 50% preferring implants over other options due to their durability. This is consistent with findings from other research, which suggests that increased knowledge about the long-term functional and aesthetic benefits of implants leads to more favorable patient attitudes [15, 16 & 17]. The literature further supports that effective patient education can reduce concerns about surgical procedures and recovery, which 35% of participants in this study cited as deterrents [18]. Studies have shown that addressing these concerns through comprehensive pre-surgical counselling can improve patient comfort and willingness to undergo dental implant procedures [19].

## Literature comparison:

Several studies have highlighted similar patterns regarding patient awareness and perceptions of dental implants. A survey by Pommer *et al.* (2011) reported that although 72% of patients were aware of dental implants, only 28% were knowledgeable about the surgical process and required maintenance, comparable to the findings of this study [20]. Similarly, a study by Zimmer *et al.* (1992) noted that the cost of implants is often perceived as a significant hurdle, with over 50% of respondents citing expense as a primary reason for not pursuing implant treatment [10]. This reinforces the notion that while awareness is high, financial concerns and lack of understanding about the full benefits and payment options continues to restrict the adoption of implants. Moreover, the correlation between positive attitudes and heightened awareness observed in this study is supported by studies like those by Tepper *et al.* (2003), which demonstrated that patients who are well-informed about the durability, aesthetics and functionality of implants are more likely to opt for them despite cost concerns [21]. After tepper add arora *et al.* in 2022 [22]. According to madhuri *et al.* in 2023" Only a small percentage of individuals had implants and more than half knew nothing about them" [23]. Low level of Knowledge was observed according to mously *et al.* in 2024 [24].

In conclusion, while awareness of dental implants is fairly widespread, significant gaps in knowledge about the procedure and cost continue to affect patient decisions. Addressing these gaps through educational initiatives that focus on both the clinical and financial aspects could lead to higher acceptance and adoption of dental implants as the preferred teeth replacement option.

## Conclusion:

Increased awareness and educational efforts are needed to address cost concerns and misconceptions about dental implants. Dental professionals should highlight financing options and long-term benefits to enhance patient uptake.
